# Loss of memory CD4^+^ T-cells in semi-wild mandrills (*Mandrillus sphinx*) naturally infected with species-specific simian immunodeficiency virus SIVmnd-1

**DOI:** 10.1099/vir.0.059808-0

**Published:** 2014-01

**Authors:** Edward J. D. Greenwood, Fabian Schmidt, Florian Liégeois, Ivanela Kondova, Anaïs Herbert, Barthelemy Ngoubangoye, François Rouet, Jonathan L. Heeney

**Affiliations:** 1 University of Cambridge, Department of Veterinary Medicine, Cambridge CB3 0ES, UK; 2 Centre International de Recherches Médicales de Franceville, Laboratoire de Rétrovirologie, Franceville, BP769, Gabon; 3 Institut de Recherches pour le Développement, UMI 233, Montpellier, BP64501, France; 4 Biomedical Primate Research Centre, Division of Pathology and Microbiology, Rijswijk 2288 GJ, The Netherlands; 5 Centre International de Recherches Médicales de Franceville, Centre de Primatologie, Franceville, BP769, Gabon; 6 Institut Pasteur du Cambodge, Unité VIH/Hépatites, 5 Boulevard Monivong, BP 983 Phnom-Penh, Cambodia

## Abstract

Simian immunodeficiency virus (SIV) infection is found in a number of African primate species and is thought to be generally non-pathogenic. However, studies of wild primates are limited to two species, with SIV infection appearing to have a considerably different outcome in each. Further examination of SIV-infected primates exposed to their natural environment is therefore warranted. We performed a large cross-sectional study of a cohort of semi-wild mandrills with naturally occurring SIV infection, including 39 SIV-negative and 33 species-specific SIVmnd-1-infected animals. This study was distinguished from previous reports by considerably greater sample size, examination of exclusively naturally infected animals in semi-wild conditions and consideration of simian T-lymphotropic virus (STLV) status in addition to SIVmnd-1 infection. We found that SIVmnd-1 infection was associated with a significant and progressive loss of memory CD4^+^ T-cells. Limited but significant increases in markers of immune activation in the T-cell populations, significant increases in plasma neopterin and changes to B-cell subsets were also observed in SIV-infected animals. However, no increase in plasma soluble CD14 was observed. Histological examination of peripheral lymph nodes suggested that SIVmnd-1 infection was not associated with a significant disruption of the lymph node architecture. Whilst this species has evolved numerous strategies to resist the development of AIDS, significant effects of SIV infection could be observed when examined in a natural environment. STLVmnd-1 infection also had significant effects on some markers relevant to understanding SIV infection and thus should be considered in studies of SIV infection of African primates where present.

## Introduction

Over 40 different African primate species are naturally infected with a species-specific simian immunodeficiency virus (SIV). Studies into the outcome of infection are limited to a few species, with SIV infection of African green monkeys (*Chlorocebus* spp.) and sooty mangabeys (*Cercocebus atys*) studied extensively, in addition to more limited examination of SIV infection of mandrills (*Mandrillus sphinx*). Such studies have demonstrated the apparent convergent evolution of multiple mechanisms to prevent the development of SIV-induced disease in these ‘natural hosts’ of SIV, despite high levels of viral replication throughout the chronic phase of infection (recently reviewed by Chahroudi *et al.*, 2012).

Features common to all three of these species include an extremely low prevalence of CD4^+^ cells bearing CCR5 (the principle co-receptor for SIV) (Paiardini *et al.*, 2011; Pandrea *et al.*, 2007a), a large population of T-cells that are CD4^–^, but may be able to carry out functions borne normally by CD4^+^ cells (Beaumier *et al.*, 2009; Milush *et al.*, 2011; Murayama *et al.*, 1999; Vinton *et al.*, 2011), limited vertical transmission of SIV and suppression of immune activation after the acute stage of infection (Bosinger *et al.*, 2009; Kornfeld *et al.*, 2005; Onanga *et al.*, 2002). In African green monkeys and sooty mangabeys, it has also been demonstrated that the gut mucosal barrier remains intact in SIV-infected animals (Gordon *et al.*, 2007; Pandrea *et al.*, 2007b). These mechanisms appear to be highly successful, as AIDS-like clinical signs are observed extremely rarely in the captive populations studied (Pandrea *et al.*, 2001, 2009). It therefore appears that in the hundreds of thousands (Ma *et al.*, 2013), if not millions (Gifford *et al.*, 2008) of years of virus–host co-evolution, a nearly perfect virus–host equilibrium has been achieved, where high levels of viral replication can occur without ill effects to the host.

However, the importance of studying such virus–host interactions in the wild has been indicated by study of SIVcpz-infected wild chimpanzees (Keele *et al.*, 2009). When studied in the wild, SIVcpz-infected animals had a greater risk of mortality, with evidence of CD4^+^ T-cell depletion, immune activation and the development of an AIDS-like disease in one animal (Keele *et al.*, 2009). By contrast, the few naturally infected chimpanzees housed in centres in the USA and Europe seemed to suffer no ill effects of infection (Gilden *et al.*, 1986; Weiss & Heeney, 2009). As a result, SIVcpz infection of chimpanzees was erroneously thought to be non-pathogenic for over a decade. A clear picture of the true effect of SIV infection therefore requires study in the natural environment.

Significant work has been published recently that helps close this gap in our knowledge. Ma *et al.* (2013) found that wild SIV-infected African green monkeys do not show evidence of chronic immune activation (by measurement of plasma cytokines) nor do they have increased soluble CD14 (sCD14), a marker upregulated where the gut mucosal barrier is compromised. However, due to the nature of their sampling of wild animals, certain analyses were not possible in this study, including characterization of lymphocyte subsets and expression of immune markers on these cells – fundamental aspects of our understanding of human immunodeficiency virus type 1 (HIV-1) infection of humans. In addition, it was not possible to observe longitudinal effects of SIV infection.

In 1983, a colony of mandrills was established at the International Centre for Medical Research, Franceville, Gabon (Wickings, 1995). This colony is maintained in a large (10^5^ m^2^) area of dense, natural rainforest and currently includes >200 mandrills. At least one animal introduced into this colony was infected with species-specific SIVmnd-1, which has spread within the colony. An analysis of the history of the spread of SIV within this colony and the likely mechanisms for this spread has been published recently (Fouchet *et al.*, 2012).

This colony provides long-term monitoring possibilities, with the possibility to capture animals for veterinary examination and sampling. It provides an ideal situation to address the outcome of SIV infection of a primate species in their natural environment. It also provides a valuable opportunity to study the outcome of natural SIV infection, avoiding the potential artefacts raised by abnormal routes of infection or challenge material. We therefore investigated a large cross-section of uninfected and naturally SIVmnd-1-infected animals to investigate the effect of natural SIVmnd-1 infection on this apparently well-adapted host, when studied in an environment representative of that found in the wild.

## Results

### Study cohort and viral loads

**Fig. 1.**
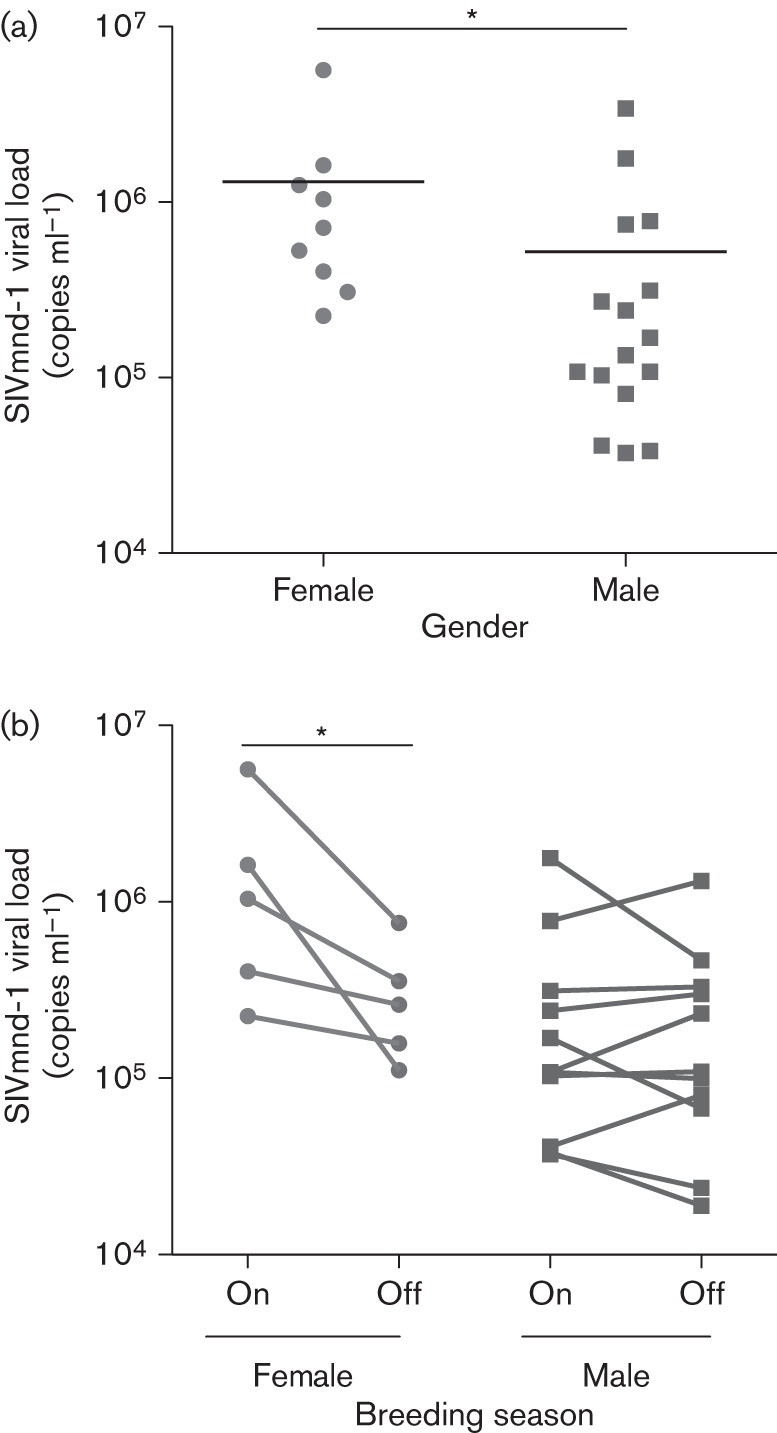
SIVmnd-1 plasma viral loads. (a) Comparison between the viral loads of males (*n* = 16) and females (*n* = 9) within this study cohort. *Gender was a significant predictor variable in a linear model of viral load including age and STLV status as covariables, with *P*<0.05. (b) Comparison of viral loads between those measured during this study (‘On’ breeding season) and those measured in the last 2 years outside the breeding season (‘Off’ breeding season) in males (*n* = 11) and females (*n* = 5). *P<0.05 in a two-tailed paired *t*-test.

The study cohort is described in Table 1. The dynamics of SIVmnd-1 and simian T-lymphotropic virus (STLV) infection in this cohort prevented precise age- and gender-matching of the groups. Age and gender were therefore included as covariates in all statistical tests throughout this work. Experimentally infected animals and SIVmnd-2-infected animals were excluded from study (and SIV therefore refers to SIVmnd-1 throughout). In addition, only chronically infected animals were included, by the criterion of known seroconversion at a prior date. The most recently infected animals were found to have seroconverted ~6 months prior to this study, while the longest duration of SIV infection was 14.9 years. One SIV-positive animal excluded from the study due to testing SIV-seronegative at the last sampling date was noted to have a high viral load and unusually high levels of immune activation, suggestive of acute infection. As it has recently been suggested that high levels of acute-phase immune activation in SIV-infected African primates may be an artefact of experimental infection (Ma *et al.*, 2013), data for this animal are presented in Fig. S1 (available in JGV Online). Analysis of plasma SIV viral loads indicated that STLV co-infection did not have a significant impact on SIV viral load (*P* = 0.7), nor did time since first SIV seroconversion (*P* = 0.3) or age (*P* = 0.8) in a general linear model. Interestingly, gender was a significant predictor of viral load (Fig. 1a), with apparently much higher levels of viral replication in females.

**Table 1.** Demographics of the semi-wild mandrill study cohort: age, gender makeup and (where relevant) SIVmnd-1 plasma viral loads and duration of SIVmnd-1 infection

Group	*n*	Age (years)			Gender (% male)	SIVmnd-1 viral load (log copies ml^−1^)				Duration of SIV infection (years)		
Minimum	Mean	Maximum	*n*	Minimum	Mean	Maximum	Minimum	Mean	Maximum
Uninfected	31	5.0	10.2	23.6	32.3							
STLV only	8	7.5	12.1	22.5	37.5							
SIVmnd-1 only	11	7.5	9.5	12.7	54.5	7	5.01	5.98	6.53	0.44	4.53	8.5
SIVmnd-1+STLV	22	8.6	14.0	20.7	68.2	18	4.57	5.87	6.75	1.57	4.86	14.87

Noting that our study (including all immunological analyses described below) was carried out at in late September and the beginning of October, which corresponds to the end of the peak mandrill breeding season (July to September) (Setchell *et al.*, 2005), we considered that hormonal changes in females might lead to minor immunosuppression and greater viral replication. We therefore identified animals that had been sampled in the last 2 years outside of the breeding period (i.e. January–May) and determined the viral load for these samples. Viral load was significantly lower in females when sampled outside the breeding period; no such relationship between breeding season and viral load was found in males (Fig. 1b).

### SIVmnd-1 infection is associated with a progressive loss of CD4^+^ memory T-cells

**Fig. 2.**
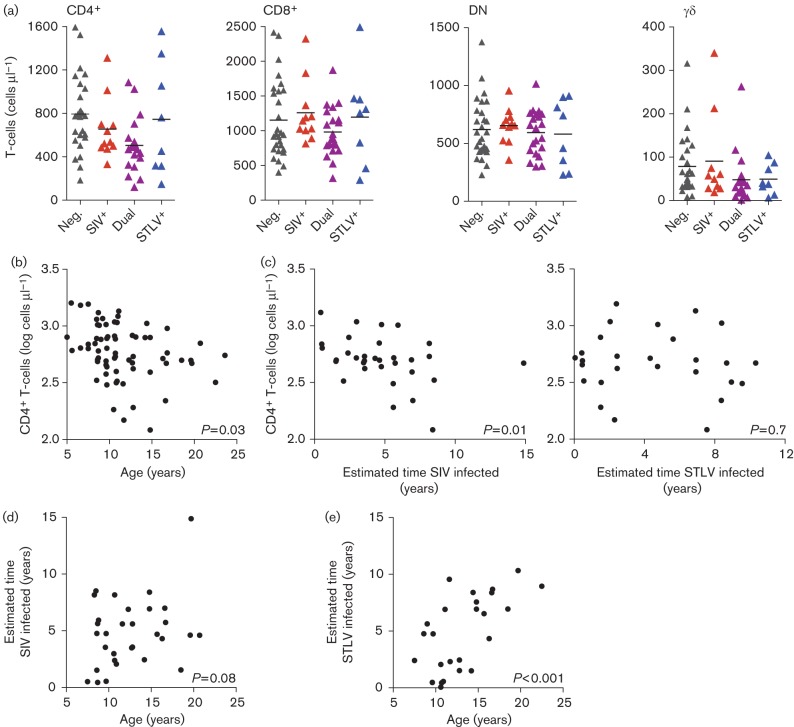
T-cell subsets in the mandrill cohort. (a) Absolute count of peripheral blood T-cells of the CD4^+^, CD8^+^, DN and γδ T-cell populations in uninfected (Neg.), SIVmnd-1-infected (SIV^+^), STLVmnd-1-infected (STLV^+^) and SIV/STLV-co-infected animals (Dual). (b) Age was associated negatively with absolute CD4^+^ T-cell count in a linear model including gender, SIV and STLV infection as additional covariates. (c) Within the SIVmnd-1-positive population, duration of SIVmnd-1 infection was significantly correlated inversely with absolute CD4^+^ T-cell number in a linear model that included age, gender and STLV infection status. No such correlation was found with duration of STLV infection in a linear model that included age, gender and SIV status. (d, e) Duration of SIV infection showed a positive correlation with age that did not reach significance (d), whilst duration of STLV infection was correlated strongly with age (e), in a linear regression analysis of duration of infection and age only. Animal numbers: Neg., *n* = 28; SIV^+^, *n* = 11; STLV^+^, *n* = 8, Dual, *n* = 21; except γδ T-cell measurements: Neg., *n* = 27; SIV^+^, *n* = 10.

Absolute numbers of peripheral blood T-cells were determined by flow cytometry, with no significant differences found in amounts of CD4^+^, CD8^+^, double-negative (DN) or γδ T-cells due to SIV or STLV infection (Fig. 2a). As found previously for mandrills and other primate species (Apetrei *et al.*, 2011; Taaffe *et al.*, 2010), there was an inverse correlation between age and absolute CD3^+^CD4^+^ count (Fig. 2b). However, there was also a significant inverse correlation between the absolute number of CD4^+^ T-cells and the time since the animal first tested positive for SIV (Fig. 2c) in a linear model that included age as a covariate. Whilst it could be argued that age and duration of infection are not independent (as older animals are likely to be infected for longer), such a correlation between CD4^+^ T-cell count was not found with duration of STLV infection (Fig. 2c), despite the fact that duration of STLV infection was a far better correlate with age of the animal than duration of SIV infection (Fig. 2d).

**Fig. 3.**
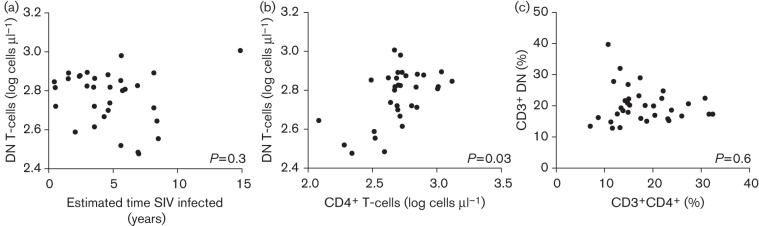
Loss of CD4^+^ T-cells is unlikely to be caused by downregulation of CD4. (a, b) Duration of SIV infection was not correlated positively with the number of DN T-cells (a), whilst absolute numbers of CD4^+^ and DN T-cells were correlated positively in the SIV-infected population (b). (c) There was no significant relationship between the percentage that CD3^+^CD4^+^ cells and CD3^+^ DN cells make up of the total lymphocyte population in the SIV-positive animals. *P*-values are the significance of the *x*-axis variable as a predictor of the *y*-axis variable in a linear model that included age, gender and STLV status.

There was no positive correlation between duration of SIV infection and the absolute DN T-cell count (Fig. 3a), a positive correlation between absolute number of CD4^+^ and DN T-cells in SIV-infected animals (Fig. 3b), and no significant inverse correlation between the percentage that these cell types represent of the total lymphocyte population (Fig. 3c). These findings indicate that the loss of CD4^+^ T-cells in SIV-infected animals was not due to down-modulation of the CD4 molecule by CD4^+^ T-cells.

**Fig. 4.**
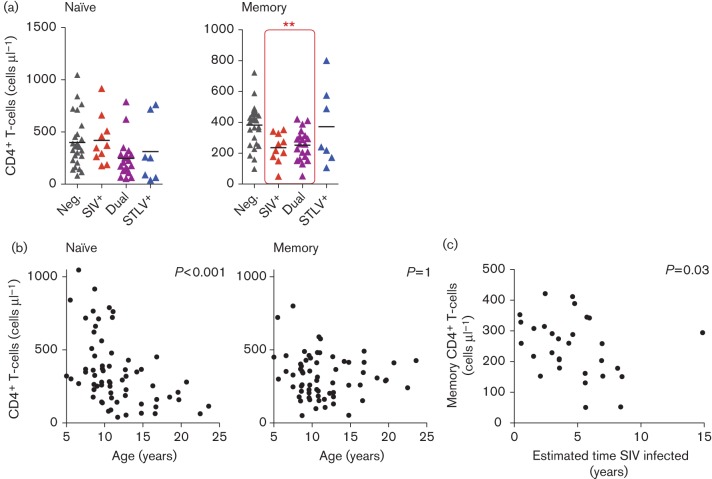
SIVmnd-1 infection is associated with progressive loss of memory CD4^+^ T-cells. (a) SIVmnd-1 infection was associated with significantly lower memory CD4^+^ T-cells, whilst no significant effect was found in naïve T-cells. **Significance of SIV as a predictor of memory CD4^+^ T-cell counts in a linear model, *P*<0.01. (b) The number of naïve CD4^+^ T-cells was strongly related inversely to age, whilst there was no relationship between age and the number of memory CD4^+^ T-cells. (c) Duration of SIVmnd-1 infection was significantly correlated inversely with absolute number of peripheral blood memory CD4^+^ T-cells. All linear models included age, gender, SIV and STLV status as predictive variables unless redundant with the subset of animals analysed. Animal numbers (see Fig. 2 for abbreviations): Neg., *n* = 28; SIV^+^, *n* = 10; STLV^+^; *n* = 7; Dual, *n* = 20.

Further examination of the naïve and memory T-cell populations within CD4^+^ T-cells, defined by CD28 and CD95 expression, revealed that SIV-infected animals had significantly fewer memory CD4^+^ T-cells (Fig. 4a). Memory T-cells were defined as the sum of the CD28^+^CD95^+^ and (infrequent) CD28^–^CD95^+^ populations. As expected, there was a significant inverse relationship between the number of naïve CD4^+^ T-cells and age, whilst there was no relationship between age and the number of memory CD4^+^ T-cells (Fig. 4b). Despite this, the number of memory CD4^+^ T-cells was significantly inversely proportional to duration of SIV infection, suggesting that SIVmnd-1 caused the progressive loss of these cells (Fig. 4c).

### Analysis of T-cell activation markers

**Fig. 5.**
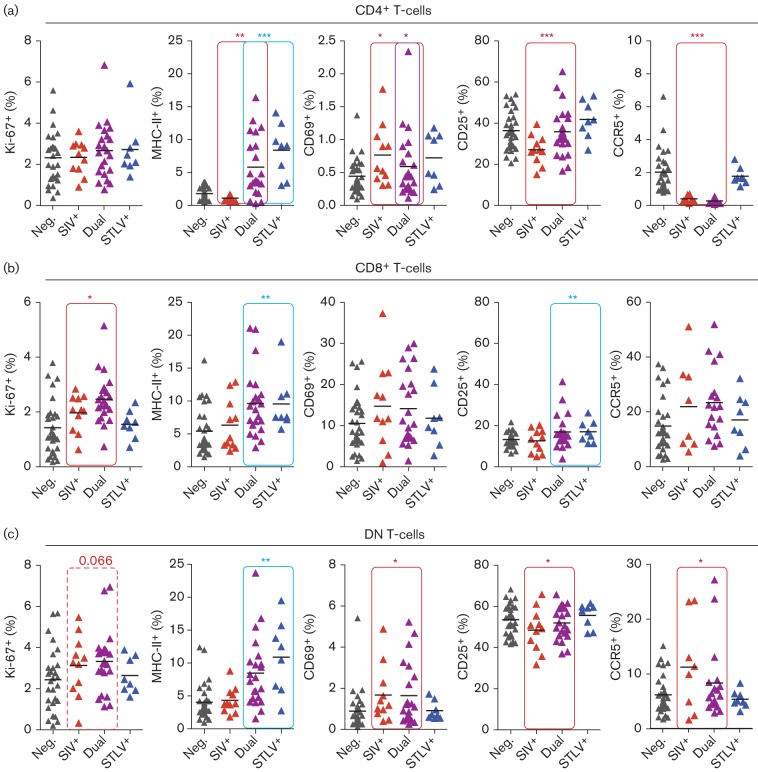
Markers of T-cell activation in SIVmnd-1 and STLVmnd-1 infection. (a–c) Percentages of CD4^+^ (a), CD8^+^ (b) and DN (c) T-cells expressing the intracellular marker Ki-67, and the cell surface markers MHC-II, CD69, CD25 and CCR5. Red and blue lines indicate where SIVmnd-1 infection and STLVmnd-1 status were significant predictors of the variable, respectively, with a purple line indicating that dual infection also had a significant effect. Effects approaching significance are indicated with a broken line and *P*-value. *P<0.05, **P<0.01, ***P<0.001. All statistical models included age, gender, SIV and STLV status as predictive variables. Animal numbers (see Fig. 2 for abbreviations): Neg., *n* = 31; SIV^+^, *n* = 11; STLV^+^, *n* = 8; Dual, *n* = 22; except CCR5 analysis: Neg., *n* = 30; SIV^+^, *n* = 8; STLV^+^, *n* = 8; Dual *n* = 18.

In order to determine whether SIVmnd-1 infection was associated with significant T-cell activation, flow cytometric analysis was carried out to assess the level of activation markers on CD4^+^, CD8^+^ or DN T-cells (Fig. 5a–c, respectively). We found a small but significant increase in the percentage of CD8^+^ T-cells expressing Ki-67 in SIVmnd-1-infected animals, with a trend for SIV to also induce increased replication of DN T-cells (*P* = 0.066). Diverse effects were seen in the expression of other markers of T-cell activation, with contradictory effects of SIV and STLV seen in some cases. STLV infection has already been demonstrated to cause upregulation of MHC class II (MHC-II) on CD4^+^ T-cells (Souquière *et al.*, 2009). We repeated this finding, also observing increased MHC-II expression in the CD8^+^ and DN population. However, we found that in the CD4^+^ population, SIV infection was associated with significantly fewer MHC-II^+^ CD4^+^ and DN T-cells.

SIV infection was also correlated with fewer CD25^+^CD4^+^ and DN T-cells. In humans, a large population of memory CD4^+^ T-cells expresses CD25 constitutively (Triplett *et al.*, 2012), and thus the lower expression of CD25 on CD4^+^ cells may reflect the loss of memory CD4^+^ T-cells described above. Highly significant loss of CCR5^+^CD4^+^ lymphocytes was also seen, as has been observed previously in sooty mangabeys (Veazey *et al.*, 2003). Finally, SIV infection induced significantly higher levels of the early activation marker CD69 on both CD4^+^ and DN T-cells.

### Soluble markers of immune activation and gut integrity

**Fig. 6.**
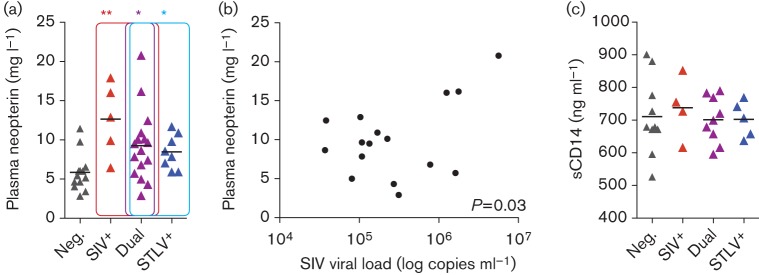
Quantification of markers of macrophage activation in plasma. (a) Concentration of plasma neopterin was quantified for a subset of the study cohort: Neg., *n* = 13; SIV^+^, *n* = 5; STLV^+^, *n* = 8; Dual, *n* = 16 (see Fig. 2 for abbreviations). (b) SIVmnd-1 was a significant predictor of plasma neopterin concentration. (c) No measurable effect of SIVmnd-1 or STLVmnd-1 on the concentration of plasma sCD14 measured was identified in a subset of the study cohort: Neg., *n* = 10; SIV^+^, *n* = 4; STLV^+^, *n* = 5; Dual, *n* = 9.

In order to investigate immune activation beyond that found in T-cells, ELISAs were used to measure the plasma concentration of soluble markers released by macrophages in response to different stimuli in a subset of the cohort. A strong and significant increase in neopterin (released specifically by macrophages in response to IFN-γ) in SIV-infected animals was found, with a smaller, but also significant increase in STLV-infected animals (Fig. 6a). Curiously, neopterin levels were reduced significantly in SIV-infected animals where STLV was also present. The amount of plasma neopterin found was correlated positively with viral load in SIV-infected animals, suggesting that the increase in neopterin identified was driven by SIV replication (Fig. 6b). In contrast, sCD14, produced by macrophages in response to lipopolysaccharide, was not found in greater concentrations in the SIVmnd-1-infected animals (Fig. 6c)

### Identification and quantification of B-cell subsets and NK cells

**Fig. 7.**
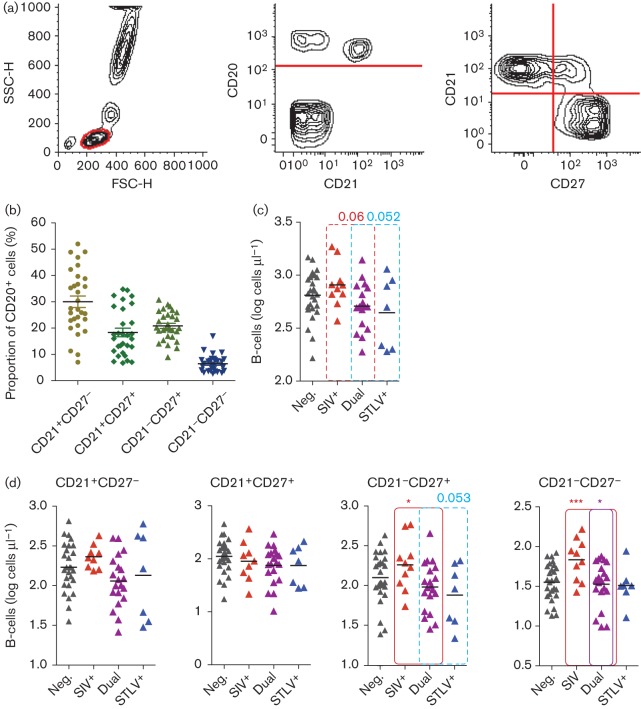
SIVmnd-1 infection was associated with significant changes in B-cell populations. (a) B-cells were defined as CD20^+^ lymphocytes, with four subsets defined by CD21 and CD27 expression. FSC-H, forward scatter height; SSC-H, side scatter height. (b) Makeup of the CD20^+^ population by CD21 and CD27 expression in uninfected animals. (c) Absolute CD20^+^ cell counts of the mandrills in this study cohort. (d) SIVmnd-1 infection was associated with significant expansion of the CD27^–^CD21^+^ and CD27^–^CD21^–^ populations. Animal numbers (see Fig. 2 for abbreviations): Neg., *n* = 27; SIV^+^; *n* = 9, STLV^+^; *n* = 7; Dual, *n* = 20.

Pathogenic HIV-1 infection of humans and SIV infection of rhesus macaques are associated with disruption of B-cell populations, but have not yet been analysed in non-pathogenic SIV infections. We quantified total, naïve and memory B-cell populations as described previously for humans and rhesus macaques (Titanji *et al.*, 2010), using a combination of CD20, CD21 and CD27 (Fig. 7a). Percentages of the four subsets in the SIV- and STLV-negative animals are displayed together in Fig. 7(b) to indicate the normal makeup of the B-cell population. When the entire study cohort was analysed, there was a trend approaching significance for SIV-infected animals to have a higher absolute number of total B-cells (*P* = 0.06) (Fig. 7c) and for STLV-infected animals to have a lower number (*P* = 0.052). Within the B-cell subsets defined here, the CD21^+^CD27^–^ population (identified as naïve B-cells in other species) was not altered in size by SIV or STLV infection (Fig. 7d). Consistent with this population representing naïve cells in this species, there was a significant inverse relationship between number of these cells and age (*P* = 0.02, not shown). SIV infection had no effect on the quantity of CD21^+^CD27^–^ or CD21^+^CD27^+^ cells. However, SIV infection was associated with a significant expansion of both CD21^–^ populations. Surprisingly, these alterations to the B-cell population were reduced significantly in STLV-infected animals compared with animals infected with SIVmnd-1 only.

In addition, NK cells were defined and analysed for the first time in mandrills. NK cells were defined as CD3^–^CD20^–^ NKp46^+^. These cells homogeneously expressed CD8, with variable CD16 expression. We found no significant changes in absolute NK cell number, or expression of CD16 related to SIV or STLV infection, although there was a trend for increased CD16 expression on NK cells in STLV-infected animals that approached significance (*P* = 0.059) (Fig. S2).

### Histological analysis of peripheral lymph nodes

In order to establish the effect of SIV infection on the architecture of secondary lymphoid tissues, histological examination of peripheral inguinal lymph nodes from uninfected (*n* = 7), SIVmnd-1-infected (*n* = 11), STLV-infected (*n* = 5) and dual-infected animals (*n* = 6) was carried out. Animals in all groups exhibited a high frequency of reactive lesions, including basic patterns (follicular, paracortical, sinusoidal) or mixed patterns with varied severity, with no apparent trend depending on retroviral infection status. Unusual collagen deposition was noted only in a single SIVmnd-1/STLV-co-infected animal, with mild deposition in the zones surrounding the high endothelial venules and the reticulin network of fibres in the subcapsular sinuses.

## Discussion

We demonstrate here that natural SIVmnd-1 infection of mandrills, studied in an environment representative of the wild, is associated with loss of memory CD4^+^ T-cells, increased levels of some markers of T-cell activation, increased plasma neopterin and perturbations to the B-cell population.

This is not the first examination of SIV infection in the International Centre for Medical Research colony. Most recently, Apetrei *et al.* (2011) investigated a smaller number of mandrills. They studied SIVmnd-1-infected, SIVmnd-2-infected and dual-infected animals, finding no significant difference in any marker analysed between uninfected and SIVmnd-1-infected animals. However, their group size for SIVmnd-1 infection (*n* = 8) was considerably smaller than ours, a possible explanation as to why they found no differences in analyses shared between their study and ours, such as the frequency of Ki-67^+^ CD8^+^ T-cells. In addition, our investigation into immune activation is broader, with a number of significant differences found in markers not included in their study. Few naturally SIVmnd-1-infected animals were included in their study (*n* = 4), whilst we examined exclusively naturally infected animals and animals housed exclusively in the semi-wild enclosure. The STLV status of animals in each group of their study was also not reported. Finally, given the high viral loads found in females in our study, which we suspect was due to immunosuppressive effects of reproductive hormones, it is possible that effects of SIVmnd-1 infection were more muted in Apetrei *et al.* (2011), assuming it was carried out at a different time of year.

We recognize that this is not the first report of progressive loss of CD4^+^ T-cells in natural SIV infection of an African primate species. Taaffe *et al*. (2010) reported that over a 5-year period, SIV-infected sooty mangabeys at the Yerkes National Primate Research Center (USA) displayed CD4^+^ loss over time that is significantly greater than the loss seen in uninfected animals. However, the authors find a significant correlation between age and CD4^+^ T-cell loss, note that the SIV-infected group is significantly older, but then do not state if the apparent faster loss of CD4^+^ T-cells in SIV-infected animals would retain significance if age was considered as a covariate – indeed, they propose that the difference in age might explain the observed phenomenon. In addition, they were unable to show a loss of either naïve or memory CD4^+^ T-cells beyond the expected changes over time also seen in the uninfected population. By contrast, the trend we have identified in semi-wild mandrills, for loss of CD4^+^ over duration of infection, was significant in a model that included age as a covariate. In addition, we were able to show that memory CD4^+^ T-cells specifically are lost in SIVmnd-1-infected animals.

We identified a pattern of small but significant increases in chronic T-cell activation induced by SIV and to a lesser extent STLV. SIV-infected mandrills had an up to 2.2-fold increase in Ki-67 expression in CD8^+^ T-cells compared with uninfected animals. This is therefore below the increase in these cells of two- to fourfold in chronically HIV-1-infected humans (Sachsenberg *et al.*, 1998) and the two- to fourfold increase observed in chronically SIVmac-infected rhesus macaques (Kaur *et al.*, 2000). In the CD4^+^ T-cell compartment, we observed significantly higher expression of the early activation marker CD69, but significantly fewer MHC-II^+^ cells. It is tempting to speculate that this was due to CD4^+^ T-cells being removed by SIV infection after the early stages of activation.

Neopterin, one of the first markers of immune activation to be identified as elevated in HIV-1 infection of humans and a predictor of disease progression (Fuchs *et al.*, 1989; Lambin *et al.*, 1986), has thus far (to our knowledge) been neglected as a marker of interest in studies of the key models of SIV infection of ‘natural host’ species. It is therefore unclear at this point whether the significant and substantial increase in neopterin identified here was found only due to the semi-wild study environment or whether it is also a feature of the well-established models of SIV infection of African primates in captive animals. Notably, whilst the T-cell activation associated with SIV infections described here was muted compared with that found in HIV-1-infected humans, the increases in neopterin induced by species-specific SIVmnd-1 and STLVmnd-1 reported here were of a similar magnitude to the increases demonstrated in HIV-1- and human T-lymphotropic virus type 1 (HTLV-1)-infected humans (Ali *et al.*, 1992; Lambin *et al.*, 1986). Neopterin is produced by macrophages in response to IFN-γ, the source of which remains unidentified in this model.

Alterations to the B-cell subsets have not been reported previously in African primate species. The changes demonstrated here must be interpreted with caution, as rigorous determination of the functions of the B-cell subsets defined by CD21 and CD27 was not within the scope of this study. However, it is tempting to draw parallels between the phenomena observed here and the accumulation of CD21^–^ ‘exhausted’ cells observed in HIV-1 infection of humans (Moir *et al.*, 2008). Further work is therefore required to characterize the nature of these changes.

Whilst we see no significant disruption of the lymph node architecture in SIVmnd-1-infected animals, reactive patterns were found frequently in all animals, further suggesting that animals in this population have a high exposure to other infectious agents, mirroring the situation in truly wild primates. The finding of mild collagen deposition in only a single animal suggests that this type of disruption of lymph node architecture, found in both HIV-1-infected humans (Schacker *et al.*, 2002) and in wild chimpanzees (Keele *et al.*, 2009), is not present in this model.

The findings of our study also differ somewhat from recently published findings on wild African green monkeys, in which no evidence of immune activation was found (Ma *et al.*, 2013). However, we note that in the one analysis shared between the two studies, our finding is the same – a lack of increased plasma sCD14. It therefore appears that in both species, even in the wild, where the mucosal barrier is presumably under greater stress from other pathogens able to disrupt it, SIV infection does not cause the catastrophic loss of integrity of this barrier found in pathogenic infections. The limited immune activation found in this study of mandrills must therefore have another cause. Our study also raises some points for consideration for the ongoing work examining African green monkeys in their natural environment (e.g. consideration of breeding season in the analysis of viral load, the effects of STLV co-infection, and the utility of determining plasma neopterin concentration as a sensitive and relevant measure of immune activation in retroviral infection).

Of note to future studies, we demonstrate here that several markers relevant to understanding the outcome of SIV infection were also affected significantly by STLV-1 infection. This includes markers of interest that were upregulated by STLV and, surprisingly, some analyses where STLV co-infection appears to ameliorate the phenotype induced by SIV. We note that STLV co-infection does not appear to be considered in the key studies of wild primates (Keele *et al.*, 2009; Ma *et al.*, 2013) nor is STLV status reported in the many studies of the outcome of SIV infection of sooty mangabeys at the Yerkes National Primate Research Centre (Silvestri *et al.*, 2003; Sumpter *et al.*, 2007; Taaffe *et al.*, 2010), despite the high prevalence of STLV-1 in this colony (Traina-Dorge *et al.*, 2005). We suggest that identifying the effect of STLV infection may be useful in future attempts to measure the consequences of SIV infection. Whilst many key parameters measured here were not affected by STLV-1 infection (e.g. only two markers of T-cell activation were affected), it would be inappropriate to predict the effect of STLV infection on such markers in other species based on these results. In addition, the study of SIV infection of sooty mangabeys includes multiple analyses that are beyond the scope of this investigation into mandrills, for which no data on the effect of STLV infection exists in any species.

As a final consideration, we note that in addition to multiple mechanisms for preventing the development of AIDS, African green monkeys, sooty mangabeys and mandrills have also developed strong barriers to vertical transmission of SIV infection, which appears to be rare in these species (Chahroudi *et al.*, 2012; Pandrea *et al.*, 2008). It is unclear as to why there should have been selection towards this phenotype if SIV infection is not detrimental. We also note that whilst rare, AIDS-like cases have been described for all three of these species (Pandrea *et al.*, 2009), in animals infected for a long period of time. In mandrills, the only case of an AIDS-like syndrome associated with SIVmnd-1 infection was described in an animal held in the semi-wild enclosure aged ~20 years old and SIV-infected for at least 17 years (Pandrea *et al.*, 2001). Given that previous reports have found no effect of chronic SIVmnd-1 infection on CD4^+^ count or immune activation (Apetrei *et al.*, 2011; Pandrea *et al.*, 2003), it is unclear if the development of AIDS in this case was due to the protracted length of SIV infection or instead due to some specific susceptibility of this animal to disease. In this study, we find that SIVmnd-1 infection of mandrills held in the semi-wild enclosure is associated generally with significant loss of memory CD4^+^ T-cells, moderate increases in activation of both T-cells and macrophages (indicated by plasma neopterin), and for the first time in a ‘natural host’ species, changes in the makeup of the B-cell population. We propose that these observations lend weight to a theory that SIVmnd-1 infection has a general potential to cause disease in mandrills exposed to their natural environment, given sufficient duration of infection, but the slow rate of damage to the immune system, combined with the paucity of vertical transmission, prevents the development of disease in most animals.

## Methods

### 

#### Ethics statement.

This study was carried out in strict accordance with European Directive CEE 10/63. The protocol was approved by the Committee on the Ethics of Animal Experiments of the Department of Veterinary Medicine, University of Cambridge (permit no. CR54). Blood draws and excision of inguinal lymph nodes were carried out under ketamine/HCl [10 mg (kg body weight)^−1^] anaesthesia. All efforts were made to minimize animal suffering.

#### Animals.

Animals included in the study were adult or late adolescents, >5 years old for females and >7.5 years for males (on average mandrills reach adult body mass at age 5 years in females and age 8 years in males; Wickings & Dixson, 1992).

#### Serological screening.

Plasma was tested for the presence of SIV antibodies with an in-house strain-specific ELISA-based assay using SIVmnd-1 lineage-specific V3-loop as antibody capture antigens (Simon *et al.*, 2001). STLV-1 was confirmed as described previously (Souquière *et al.*, 2009). Briefly, plasma was screened for cross-reactive antibodies to HTLV-I/II by ELISA (HTLV-I Platelia New, Bio-Rad or Vironostica, bioMérieux). STLV-1 infection was confirmed by Western blotting (HTLV blot 2.4; Diagnostic Biotechnology) or InnoLIA HTLV-I/II strip assay (Innogenetics).

#### SIVmnd-1 viral load.

Viral load assays were performed as described previously (Liégeois *et al.*, 2012). Briefly, the assay is an integrase-based real-time PCR assays using a minor groove binder probe specific of SIVmnd-1. For the quantitative PCR standard, a virus-specific plasmid was used (Invitrogen). Primers: AGTAAGTGCTGTCATCATGGAGA; GTTTCTGCTGTTATAACTTCTGCCTTC. Probe: AGATGCTTCACTTGGAGTC.

#### Flow cytometric analysis.

For staining of surface markers, 100 µl whole blood was incubated with a mAb mixture in a 5 ml polystyrene round-bottomed tube (Falcon 2058; BD) at room temperature for 15 min. Antibodies were selected on the basis of cross-reactivity and were titrated for this species. After this first incubation, 1.5 ml FACS lysing solution (BD) was added to all tubes, followed by incubation at room temperature for 15 min and then centrifugation for 5 min at 500 ***g***. The supernatant was aspirated [with the exception of tubes with counting beads (AccuCheck; Invitrogen), which were directly acquired], and cells were washed with 1 ml PBS and centrifuged for 5 min at 500 ***g***. This washing step was finished by aspiration of the majority of the supernatant followed by data collection on a FACScalibur (BD). Intracellular Ki-67 staining was carried out with a slight variation on this procedure. After lysis, centrifugation and removal of supernatant, cells were resuspended in 1× Perm/Wash solution (BD) containing fluorescently conjugated anti-Ki-67 and incubated for 30 min at room temperature in the dark before a wash with 1× Perm/Wash solution and acquisition. Data were analysed with FlowJo v9 (Tree Star). Compensation was carried out post-acquisition based on data for single-stained cells acquired in parallel. Group sizes varied for flow cytometric analysis: pre-acquisition, some animals were excluded due to limited availability of materials; post-acquisition, absolute count data were excluded from animals that did not pass the AccuCheck internal quality control. Antibodies against the following markers were used, with clone names in parentheses: CD3ϵ (SP34.2; BD), CD3γδ (B1.1), CD4 (SK3), CD8 (SK1), CD16 (3G8), CD20 (L27; BD), CD21 (B-ly4; BD), CD25 (4e3; Mulitenyi), CD27 (M-T247; BD), CD28 (CD28.2), CD69 (FN50), CD95 (DX2), MHC-II (L243), Ki-67 (B56) and CCR5 (3A9; BD). Antibodies were supplied by Ebioscience, unless specified.

#### Analysis of plasma soluble markers.

Commercial ELISA kits were used to test for plasma concentration of neopterin and sCD14, supplied by IBL International and Abcam, respectively. Assays were carried out according to the manufacturer’s instructions.

#### Histological analysis of inguinal lymph nodes.

Excision lymph node biopsies were collected in 10 % saline-buffered formalin and embedded in paraffin. Blocks were cut into 4 µm sections on to standard glass slides, stained using haematoxylin & eosin and Masson’s trichrome, and examined by light microscopy. Histological examination was undertaken without knowledge of infection status.

#### Statistical analysis.

The r package (http://www.r-project.org/) was used for the majority of the statistical analysis. General linear models were used to determine if SIV and STLV infection had a significant effect on the response variable in question. Age, gender, and SIVmnd-1 and STLV status were included as explanatory variables in all models, and an interaction between SIV and STLV was included where this improved model fit. Age, gender and STLV status were also retained as predictor variables when investigating the effect of duration of SIV infection or viral load within the SIV-positive group. Variable data were transformed where necessary to produce normality. Differences in viral load between the breeding and non-breeding season were analysed using a paired *t*-test in Prism 5.0 (GraphPad).
